# Supplementary material for: Transcriptomic Insights into Mechanisms of Early Seed Maturation in the Garden Pea (Pisum sativum L.)
Source: Cells. 2020 Mar 23;9(3):779. doi: 10.3390/cells9030779 (PMC7140803; doi:10.3390/cells9030779)

Zhewan−1(25)

Global Summary

%DE = 0.82

genes with fdr < 0.2 = 466 (154 + / 312 −)

genes with fdr < 0.1 = 464 (154 + / 310 −)

genes with fdr < 0.05 = 431 (137 + / 294 −)

genes with fdr < 0.01 = 346 (83 + / 263 −)

genes in genesets = 0

<FC> = 0

<t−score> = −713.17

<p−value> = 0

<fdr> = 0.18

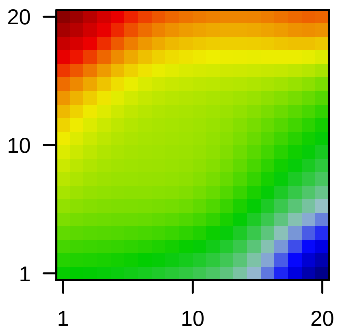

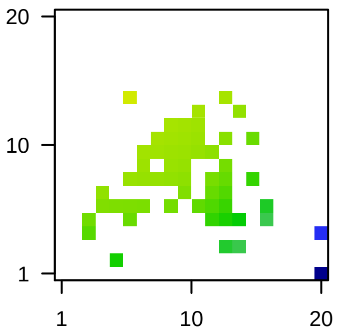

Global Genelist

Rank	ID	log(FC)	p−value	fdr	Metagene	Description
1	comp43413_c	−0.02	1e−14	8e−13	12 x 8	
2	TRINITY_DN2	−0.16	1e−14	3e−12	16 x 6	
3	TRINITY_DN4	−0.15	4e−14	3e−12	5 x 2	
4	TRINITY_DN1	−0.15	6e−14	3e−12	14 x 3	
5	TRINITY_DN4	−0.45	9e−14	1e−11	20 x 4	
6	TRINITY_DN2	−0.1	2e−13	2e−11	13 x 3	
7	TRINITY_DN7	−0.18	4e−13	2e−11	16 x 5	
8	TRINITY_DN1	−0.07	7e−13	2e−11	3 x 4	
9	comp11051_c	−0.02	7e−13	2e−10	11 x 6	
10	TRINITY_DN6	−0.05	3e−12	2e−10	6 x 6	
11	TRINITY_DN1	−0.03	6e−12	2e−10	12 x 7	
12	TRINITY_DN1	−0.02	6e−12	2e−10	10 x 7	
13	comp16243_c	−0.01	7e−12	2e−10	6 x 8	
14	comp16669_c	−0.01	8e−12	2e−10	10 x 7	
15	TRINITY_DN3	−0.03	1e−11	2e−10	11 x 6	
16	TRINITY_DN5	−0.03	1e−11	2e−10	13 x 8	
17	TRINITY_DN2	−0.04	1e−11	6e−10	12 x 6	
18	TRINITY_DN6	−0.04	2e−11	1e−09	5 x 6	
19	TRINITY_DN2	−0.11	3e−11	1e−09	15 x 8	
20	TRINITY_DN5	−0.01	5e−11	1e−09	11 x 10	

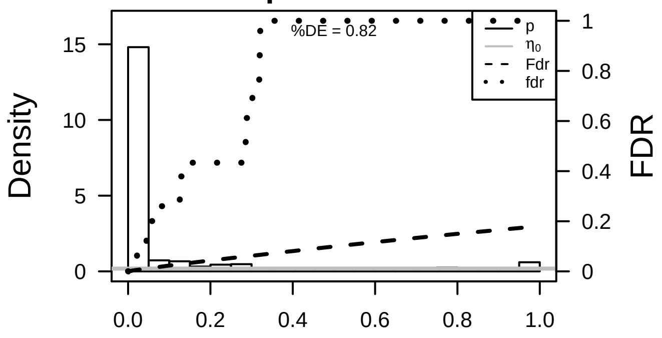

Supplement: Supplementary file 1 [file cells-09-00779-s001.zip › Supplementary materials/FileS3.zip!/FileS2/Hormones - Results/Summary Sheets - Groups/Reports/Zhewan.1.25..pdf]
